# Carbapenem-resistant *Pseudomonas aeruginosa* infections in critically ill children: Prevalence, risk factors, and impact on outcome in a large tertiary pediatric hospital of China

**DOI:** 10.3389/fpubh.2023.1088262

**Published:** 2023-02-09

**Authors:** Weichun Huang, Xiaoshu Wei, Guifeng Xu, Xingyu Zhang, Xing Wang

**Affiliations:** ^1^Department of Laboratory Medicine, Shanghai Children's Medical Center, School of Medicine, Shanghai Jiaotong University, Shanghai, China; ^2^Department of Nursing, Huashan Hospital, Fudan University, Shanghai, China; ^3^Innovation Research Institute of Traditional Chinese Medicine, Shanghai University of Traditional Chinese Medicine, Shanghai, China

**Keywords:** carbapenem-resistant *Pseudomonas aeruginosa*, pediatric, risk factor, mortality, antimicrobial resistance

## Abstract

**Background and aims:**

Carbapenem-resistant *Pseudomonas aeruginosa* (CRPA) is a major cause of healthcare-associated infections worldwide, but comprehensive study of clinical characteristics for CRPA infections among critically ill children remains limited in China. The objective of this study was to determine the epidemiology, risk factors, and clinical outcomes of CRPA infections among critically ill pediatric patients in a large tertiary pediatric hospital in China.

**Methods:**

A retrospective case-control study of patients with *P. aeruginosa* infections was conducted in the three intensive care units (ICUs) of Shanghai Children's Medical Center from January 2016 to December 2021. All patients with CRPA infection in the ICUs were enrolled as case patients. Patients with carbapenem-susceptible *P. aeruginosa* (CSPA) infection were randomly selected as control patients in a ratio of 1:1. Clinical characteristics of those inpatients were reviewed through the hospital information system. Univariate and multivariate analyses were performed to evaluate risk factors associated with the development of CRPA infections and mortality of *P. aeruginosa* infections.

**Results:**

A total of 528 cases of *P. aeruginosa* infection in the ICUs were enrolled in the 6-year study. The prevalence of CRPA and MDRPA (multidrug-resistance *P. aeruginosa*) was 18.4 and 25.6%, respectively. Significant risk factors related to CRPA infection were the length of hospitalization >28 days (OR = 3.241, 95% CI 1.622–6.473, *p* = 0.001), receiving invasive operations (OR = 2.393, 95% CI 1.196–4.788, *p* = 0.014) and a blood transfusion (OR = 7.003, 95% CI 2.416–20.297, *p* < 0.001) within 30 days before infection. Conversely, birth weight ≥2,500 g (OR = 0.278, 95% CI 0.122–0.635, *p* = 0.001) and breast nursing (OR = 0.362, 95% CI 0.168–0.777, *p* = 0.009) were significant protective factors against CRPA infections. The in-hospital mortality rate was 14.2%, and no difference in mortality was observed between patients with CRPA and CSPA infections. Platelet < 100 × 10^9^/L (OR = 5.729, 95% CI 1.048–31.308, *p* = 0.044) and serum urea <3.2 mmol/L (OR = 5.173, 95% CI 1.215–22.023, *p* = 0.026) were independent predictors for the mortality due to *P. aeruginosa* infection.

**Conclusions:**

Our findings provide insights into CRPA infections among critically ill children in China. They provide guidance in identifying patients that may be at high risk for a resistant infection and emphasize the importance of antimicrobial stewardship and infection control in hospitals.

## Introduction

*Pseudomonas aeruginosa* is one of the most common pathogens in healthcare-associated infections worldwide, causing bacteraemia, catheter-related infections, surgical site infections, and ventilator-associated pneumonia (1, 2). These infections often occur in immunocompromised or critically ill patients. In 2021, *P. aeruginosa* was the third most commonly isolated Gram-negative bacilli (GNB) in China, accounting for 7.96% of healthcare-associated infections according to China Antimicrobial Surveillance Network (CHINET) (3). In the European Union, *P. aeruginosa* (6.2%) also ranked third most common negative bacilli in 2020 (4). It has also been reported to be the second leading pathogen responsible for nosocomial pneumonia in the United States (5). Infections by *P. aeruginosa* have spread worldwide and might contribute to adverse developmental outcomes (6).

*Pseudomonas aeruginosa* is difficult to eradicate because of its remarkable ability to counter most antibiotics. On the one hand, it shows high intrinsic resistance to most β-lactams through AmpC β-lactamase, efflux pumps and outer membrane permeability; On the other hand, it possesses an extraordinary capacity to acquire resistance to nearly all other antibiotics (7, 8). In the European Union, 17.3% of *P. aeruginosa* isolates exhibited resistance to at least two or more groups of antibiotics in 2020 (4). The US CDC estimates that 32,600 healthcare-associated infections are caused by MDRPA, resulting in 707 million dollars in direct medical costs in 2017 (9). Carbapenems, a class of broad-spectrum β-lactam antibiotics, are often the last resort against treating MDRPA infections, therefore the emergence of resistance to carbapenems is challenging owing to the limited number of antimicrobial agents available to treat this type of infection (10). In Europe, carbapenem resistance is much more severe in *P. aeruginosa* (17.8%) than in *Klebsiella pneumoniae* (10%). The prevalence of carbapenem-resistant *K. pneumoniae* strains was higher in adults, whereas carbapenem-resistant *P. aeruginosa* was more frequent in children (4, 11). Previous research in the United States has revealed that the proportion of CRPA increased from 9.4% in 1999 to 20% in 2012 among children (12). The WHO has classified CRPA as a pathogen of critical priority that urgently needed novel antibiotics (13).

Serious CRPA infections are generally associated with high mortality and usually occur in patients in the ICU. These patients usually suffer from severe underlying diseases and routine invasive procedures, which are commonly associated with the occurrence and mortality of CRPA infections in many studies (14, 15). The incidence of infection in ICU patients is five–seven-fold higher than that in general inpatients, contributing to 20%−25% of all nosocomial infections in hospitals (16). It is important to timely identify risk factors for CRPA infection to prompt an effective intervention strategy to prevent the development of CRPA. Many previous studies of *P. aeruginosa* infections have focused on adult patients, especially in patients with bacteraemia (17), hematologic malignancies (18) or transplant recipients (19). So far, very little information is available on *P. aeruginosa* infections in children receiving intensive care. Given the clinical importance of CRPA infections among children, we conducted a comprehensive analysis of the epidemiology, risk factors and mortality of pediatric patients suffering from CRPA infection in the ICUs to provide insight into hospital infection control measures strategies and clinical antimicrobial treatment.

## Methods

### Study design

A retrospective case-control study was conducted in Shanghai Children's Medical Center, affiliated with Shanghai Jiaotong University, which is one of the largest and best pediatric hospitals in China with 1,500 beds. There are three ICUs in the hospital, including neonatal ICU, cardiac ICU and pediatric ICU. All patients with *P. aeruginosa* infection in the three ICUs were identified through the computerized microbiology laboratory databases of the hospital from January 1st, 2016 to December 31st, 2021. *Pseudomonas aeruginosa* infection was defined as a positive result in culture in addition to symptoms, signs, and laboratory test results indicating an infectious disease. All those infections in this study were referred to the diagnostic criteria for nosocomial infections issued by the National Ministry of Health (20).

During the study period, pediatric patients who met the inclusion criteria were enrolled in the current study. Children with acquired CRPA infection were matched in a 1:1 ratio to control patients with acquired CSPA infection during the same period. Data from all patients in this study were collected from the hospital information system. These data included patient baseline characteristics, infection characteristics, management data, microbiological data, and outcomes. The study was approved by the Ethics Committee of Shanghai Children's Medical Center (SCMCIRB-K2022127-1).

### Data collection and definitions

The demographic and clinical data from medical records included age, gender, birth weight, vaginal delivery, first child, intrapartum asphyxia, prematurity, breast nursing, clinical symptoms (fever, icterus, vomiting, diarrhea, seizures, pleural effusion, apnea), comorbidities, clinical outcome (28-day mortality, in-hospital mortality, length of hospitalization, presentation with septic shock), co-carriage with other microorganisms (bacteria, *Acinetobacter baumannii, Pseudomonas aeruginosa, Staphylococcus aureus, Escherichia coli*, and virus), medication and intervention therapy within 30 days (parenteral nutrition, previous surgery, tracheal/gastric intubation, retention catheterization, deep venipuncture catheterization, glucocorticoid/hydragogue therapy, blood transfusion and antibiotic exposure history).

Furthermore, in the laboratory examination the C-reactive protein (CRP), hemoglobin (HB), albumin (ALB), neutrophils percentage (%), lymphocyte percentage (%), platelet count (PLT), leukocytes count (WBC), alanine transaminase (ALT), aspartate aminotransferase (AST), serum urea, serum creatinine were also collected.

### Microbiology

Bacterial species identification and antimicrobial susceptibility testing were carried out on an automated Vitek-2 system (bioMerieux, France) and the AST-GN card following the manufacturer's instructions. Drug susceptibilities were interpreted in accordance with the guidelines of the Clinical and Laboratory Standards Institute M100 (21). CRPA strains were defined as *P. aeruginosa* strains that presented resistance to one of ertapenem, imipenem, or meropenem. In addition, multidrug-resistant *P. aeruginosa* (MDRPA) and extensively drug-resistant *P. aeruginosa* (XDRPA) were defined according to the criteria of international standardized terms (22).

### Statistical analysis

Data analysis was performed using SPSS 26.0 software (IBM Corporation). Patients' characteristics are generally expressed with the mean and standard deviation or median and interquartile range (IQR) for continuous variables, and with count and percentages for categorical variables. The continuous variables of descriptive statistics were tested by Student's *t*-test or Mann–Whitney *U*-test, while the categorical variables were tested by Chi-square or Fisher's exact tests. Binary logistic regression was performed to evaluate risk factors associated with the development of CRPA infections and mortality. Significant variables with *p*-value of < 0.05 were then selected into a logistic regression model for multivariate analysis to evaluate risk factors for CRPA infection and *P. aeruginosa* infection-related mortality. Results were presented as odds ratios (ORs), 95% confidence intervals (CIs), and *p*-values. All *p*-values < 0.05 with two-tailed were regarded as significant.

## Results

### Percentage of CRPA in the ICUs from 2016 to 2021

**Figure 1:**
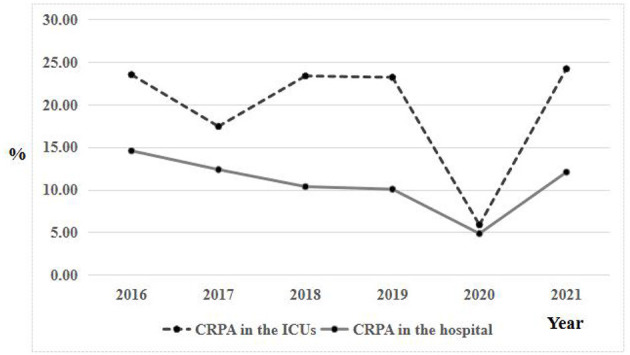
Comparison of trends in carbapenem resistance in the ICUs and all hospital wards from 2016 to 2021.

From 2016 to 2021, a total of 528 *P. aeruginosa* strains isolated from ICUs patients were enrolled in this study, of which 97 were CRPA infections and 431 were CSPA infections. The prevalence of CRPA was 18.4% (97/528), which was higher than 10.4% of all hospital inpatients. Specifically, the proportion of CRPA showed decreased but fluctuating trend from 2016 (23.5%) to 2020 (5.9%), then increased greatly in 2021 (24.2%; Figure 1). A similar trend was also observed in the proportion of CRPA in our hospital. Notably, more than 90% of CRPA strains in the hospital were isolated from ICU wards.

### Antimicrobial resistance patterns among *P. aeruginosa* isolates

The antimicrobial resistance profiles of 528 *P. aeruginosa* strains are summarized in Table 1. Of these strains, 135 (25.6%) were MDRPA and 12 (2.3%) were XDRPA. In general, these strains showed low or moderate rates of resistance to most tested antimicrobials. Specifically, the most resistant antibiotic in this study was meropenem or imipenem (18.4%), followed by aztreonam (15.6%), piperacillin (14.6%), ceftazidime (11.4%), cefoperazone/sulbactam (10.2%). For the rest antibiotics, the resistance rates were no more than 8%, including 7.5% to cefepime, 6.8% to amikacin, 5.9% to levofloxacin, 5.8% to gentamicin, 5.7% to piperacillin/tazobactam, 3.9% to ciprofloxacin, 3.2% to tobramycin and 0.6% to colistin.

**Table 1:** The antimicrobial resistance of *Pseudomonas aeruginosa* strains in this study.

**Antibiotics**	**Total** ***N* = 528** ***R* (*n*,%)**	**CSPA** ***N* = 431** ***R* (*n*,%)**	**CRPA** ***N* = 97** ***R* (*n*,%)**	***p-*Value**
**Aminoglycosides**				
Gentamicin	31 (5.8)	4 (1.0)	27 (28.2)	**< 0.001**
Amikacin	36 (6.8)	7 (1.7)	29 (29.6)	**< 0.001**
Tobramycin	17 (3.2)	2 (0.5)	15 (15.5)	**< 0.001**
**Carbapenems**				
Meropenem	97 (18.4)	0 (0.0)	97 (100.0)	**< 0.001**
Imipenem	97 (18.4)	0 (0.0)	97 (100.0)	**< 0.001**
**Cephalosporins**				
Ceftazidime	60 (11.4)	19 (4.5)	41 (42.3)	**< 0.001**
Cefepime	40 (7.5)	4 (1.0)	36 (36.6)	**< 0.001**
**Cephalosporins** + β**-lactamase inhibitors**				
Cefoperazone/sulbactam	54 (10.2)	18 (4.1)	36 (47.9)	**< 0.001**
**Fluoroquinolones**				
Ciprofloxacin	31 (3.9)	6 (1.4)	25 (25.4)	**< 0.001**
Levofloxacin	31 (5.9)	8 (1.9)	23 (23.9)	**< 0.001**
**Monobactams**				
Aztreonam	84 (15.6)	23 (5.3)	61 (63.4)	**< 0.001**
**Penicillins**				
Piperacillin	77 (14.6)	33 (7.6)	44 (45.1)	**< 0.001**
**Penicillins** + β**-lactamase inhibitors**				
Piperacillin/tazobactam	30 (5.7)	5 (1.2)	25 (25.4)	**< 0.001**
**Lipopeptide**				
Colistin	3 (0.6)	0 (0.0)	3 (3.0)	**0.006**
MDR-PA	135 (25.6)	67 (15.5)	68 (70.1)	**< 0.001**
XDR-PA	12 (2.3)	0 (0.0)	12 (12.4)	**< 0.001**

CRKP, carbapenem-resistant P. aeruginosa; CSKP carbapenem-susceptible P. aeruginosa; MDR, multidrug-resistant; XDR, extensively-drug resistant; R, resistant.

Bold values indicate significant p-values < 0.05.

Apart from imipenem or meropenem, resistance to colistin was only observed in CRPA strains (3%). CSPA exhibited low resistance rate to all antibiotics and the rate of resistance to piperacillin (7.6%) was the highest. Compared with CSPA, CRPA strains showed significantly higher rates of resistance to most tested antimicrobials, as shown in Table 1. Rates of resistance to aztreonam were much higher (63.4%). More than 40% of all strains were resistant to cefoperazone/sulbactam, piperacillin, and ceftazidime.

### Clinical characteristics of pediatric patients with *P. aeruginosa* infections

From 2016 to 2021, all 97 patients with CRPA infection in the ICUs were classified as a case group. Of the remaining 431 patients, 97 were randomly selected as the control group, with a ratio of 1:1. Finally, a total of 194 pediatric inpatients who met the inclusion criteria were included in the study.

The average age was 7 months and 60.8% were male. Among 194 patients, 110 (56.7%) patients had been breastfed and 138 (71.1%) had a birth weight of more than 2,500 g. Fever was the most common symptom (29.9%) and severe pneumonia occurred in 38 (19.6%) episodes. Anemia and congenital heart disease were observed in 26 (13.4%) and 24 (12.4%) episodes, respectively.

The statistical breakdown based on infection source was as follows: respiratory tract infection (84.0%), bloodstream infection (7.2%), urinary tract infection (3.1%) and other infections (5.2%). In the course of antibiotic treatment, the most frequently used drug was the third-generation cephalosporins (62.4%), followed by carbapenems (43.3%) and glycopeptides (33.5%).

Nearly half of the patients underwent surgery within 1 month and 102 (52.6%) patients were exposed to some sort of invasive procedures before infection confirmation. For example, 92 (47.4%) patient had tracheal intubation, 16 (8.2%) with deep venipuncture catheterization, 10 (5.2%) with gastric intubation, seven (3.6%) with retention catheterization. The baseline clinical and demographic characteristics of the entire study population are presented in Table 2.

**Table 2:** Baseline and clinical characteristics of 194 hospitalized children with *Pseudomonas aeruginosa* infections.

**Characteristics**	**Total *N* = 194**	**CSPA *N* = 97**	**CRPA *N* = 97**	***p*-Value**
**Demographics**				
Male gender	118 (60.8)	58 (59.8)	60 (61.9)	0.883
Age (days)	210 (82–735)	180 (60–615)	210 (91–960)	0.212
**Age distribution**				
< 3 months	51 (26.3)	28 (28.9)	23 (23.7)	0.514
3–5.9 months	29 (14.9)	16 (16.5)	13 (13.4)	0.688
6–11.9 months	42 (21.6)	19 (19.6)	23 (23.7)	0.601
12 months to 3 years	30 (15.5)	16 (16.5)	14 (14.4)	0.843
≥3 years	41 (21.1)	18 (18.6)	23 (23.7)	0.482
**Prematurity**	28 (14.4)	9 (9.3)	19 (19.6)	0.065
**Birth weight (g)**				
≥2,500 g	138 (71.1)	83 (85.6)	55 (56.7)	**< 0.001**
1,500–2,499 g	25 (12.9)	10 (10.3)	15 (15.5)	0.392
1,000–1,499 g	7 (3.6)	2 (2.1)	5 (5.2)	0.444
< 1,000 g	2 (1.0)	1 (1.0)	1 (1.0)	1.000
**Vaginal delivery**	102 (52.6)	58 (59.8)	44 (45.4)	0.061
**Breast nursing**	110 (56.7)	64 (66.0)	46 (47.4)	**0.014**
**First child**	97 (50.0)	53 (54.6)	44 (45.4)	0.251
**Intrapartum asphyxia**	16 (8.2)	5 (5.2)	11 (11.3)	0.191
**Specimen type**				
Sputum	163 (84.0)	86 (88.7)	77 (79.4)	0.116
Blood	14 (7.2)	5 (5.2)	9 (9.3)	0.406
Urine	6 (3.1)	4 (4.1)	2 (2.1)	0.683
Others	10 (5.2)	2 (2.1)	8 (8.2)	0.100
**Clinical symptoms**				
Fever	58 (29.9)	30 (30.9)	28 (28.9)	0.875
Icterus	17 (8.8)	12 (12.4)	5 (5.2)	0.126
Vomiting	10 (5.2)	6 (6.2)	4 (4.1)	0.747
Diarrhea	12 (6.2)	8 (8.2)	4 (4.1)	0.372
Apnea	14 (7.2)	4 (4.1)	10 (10.3)	0.163
**Complications/underlying disease**				
Severe pneumonia	38 (19.6)	14 (14.4)	24 (24.7)	0.103
Respiratory failure	14 (7.2)	4 (4.1)	10 (10.3)	0.163
Septic shock	21 (10.8)	9 (9.3)	12 (12.4)	0.645
Congenital heart disease	24 (12.4)	12 (12.4)	12 (12.4)	1.000
Hypoproteinemia	10 (5.2)	2 (2.1)	8 (8.2)	0.100
Anemia	26 (13.4)	11 (11.3)	15 (15.5)	0.528
Leukemia	15 (7.7)	7 (7.2)	8 (8.2)	1.000
**Length of hospitalization (days)**				
< 7	7 (3.6)	6 (6.2)	1 (1.0)	0.118
7–13	27 (13.9)	18 (18.6)	9 (9.3)	0.096
14–28	69 (35.6)	45 (46.4)	24 (24.7)	**0.003**
>28	91 (46.9)	28 (28.9)	63 (64.9)	**< 0.001**
**Outcome**				
28-day mortality	5 (2.6)	4 (4.1)	1 (1.0)	0.368
Hospital mortality	12 (6.2)	4 (4.1)	8 (8.2)	0.372
**Laboratory index**				
Hemoglobin < 110 g/L	86 (44.3)	47 (48.5)	39 (40.2)	0.312
Hemoglobin < 90 g/L	54 (27.8)	26 (26.8)	28 (28.9)	0.873
C-reactive protein >8 mg/L	112 (57.7)	59 (60.8)	53 (54.6)	0.468
Albumin < 35 g/L	82 (42.3)	37 (38.1)	45 (46.4)	0.309
Neutrophile >70%	70 (36.1)	31 (32.0)	39 (40.2)	0.295
Neutrophile < 50%	57 (29.4)	28 (28.9)	29 (29.9)	1.000
Lymphocyte >40%	54 (27.8)	27 (27.8)	27 (27.8)	1.000
Lymphocyte < 20%	74 (38.1)	34 (35.1)	40 (41.2)	0.460
Platelet < 100 × 10^9^/L	46 (23.7)	21 (21.6)	25 (25.8)	0.613
WBC >15.0 × 10^9^/L	46 (23.7)	25 (25.8)	21 (21.6)	0.613
ALT >69 U/L	36 (18.6)	14 (14.4)	22 (22.7)	0.196
ALT < 13 U/L	24 (12.4)	13 (13.4)	11 (11.3)	0.828
Urea < 3.2 mmol/L	58 (29.9)	30 (30.9)	28 (28.9)	0.875
Urea >7.1 mmol/L	39 (20.1)	20 (20.6)	19 (19.6)	1.000
Creatinine < 9 μmol/L	5 (2.6)	1 (1.0)	4 (4.1)	0.368
Creatinine >88 μmol/L	7 (3.6)	2 (2.1)	5 (5.2)	0.444
AST < 15 U/L	2 (1.0)	2 (2.1)	0 (0.0)	0.497
AST >46 U/L	106 (54.6)	53 (54.6)	53 (54.6)	1.000
**Pathogenies co-infections**				
*Acinetobacter baumannii*	19 (9.8)	11 (11.3)	8 (8.2)	0.630
*Klebsiella pneumoniae*	22 (11.3)	7 (7.2)	15 (15.5)	0.111
*Staphylococcus aureus*	8 (4.1)	3 (3.1)	5 (5.2)	0.721
*Escherichia coli*	5 (2.6)	3 (3.1)	2 (2.1)	1.000
Virus	10 (5.2)	3 (3.1)	7 (7.2)	0.331
**Treatments**				
**Previous antibiotic therapy (within 30 days)**				
3rd cephalosporins	121 (62.4)	63 (64.9)	58 (59.8)	0.553
Carbapenems	84 (43.3)	41 (42.3)	43 (44.3)	0.885
Glycopeptides	65 (33.5)	31 (32.0)	34 (35.1)	0.761
Aminoglycosides	23 (11.7)	12 (12.4)	11 (11.1)	0.827
Penicillins	21 (10.8)	11 (11.3)	10 (10.3)	1.000
Macrolides	23 (11.9)	12 (12.4)	11 (11.3)	1.000
Tetracyclines	9 (4.6)	4 (4.1)	5 (5.2)	1.000
Fluoroquinolones	16 (8.2)	4 (4.1)	12 (12.4)	0.065
Antifungal agents	35 (17.9)	15 (15.5)	20 (20.2)	0.457
**Blood transfusion (within 30 days)**	36 (18.6)	6 (6.2)	30 (30.9)	**< 0.001**
**Corticosteroid therapy (within 30 days)**	6 (3.1)	4 (4.1)	2 (2.1)	0.683
**Invasive operation (within 30 days)**	102 (52.6)	36 (37.1)	66 (68.0)	**< 0.001**
Tracheal intubation	92 (47.4)	33 (34.0)	59 (60.8)	**< 0.001**
Gastric intubation	10 (5.2)	4 (4.1)	6 (6.2)	0.747
Retention catheterization	7 (3.6)	2 (2.1)	5 (5.2)	0.444
Deep venipuncture catheterization	16 (8.2)	4 (4.1)	12 (12.4)	0.065
**Parenteral nutrition**	20 (10.3)	6 (6.2)	14 (14.4)	0.096
**Previous surgery (within 3 months)**	91 (46.9)	47 (48.5)	44 (45.4)	0.774
**Hospitalization expenses**	122,725 (70,536–196,748)	99,154 (54,564–149,504)	163,700 (86,153–334,968)	**< 0.001**

CRPA, carbapenem-resistant Pseudomonas aeruginosa; CSPA, carbapenem-susceptible Pseudomonas aeruginosa; ALT, Alanine transaminase; WBC, white blood cell.

Bold values indicate significant p-values < 0.05.

### Risk factors for the development of CRPA infections in the ICUs

To identify risk factors predicting the acquisition of CRPA infections in the ICUs, we conducted a retrospective case-control study that included 97 patients with CRPA infection and 97 patients with CSPA infection.

The clinical characteristics of ICUs patients with CSPA and CRPA infections are compared in Table 2. There was no difference in age and sex between these two groups. On univariate analysis, CSPA infections tend to occur in patients with birth weight ≥2,500 g (85.6 vs. 35.7%, *p* < 0.001) and receiving breast nursing (66.0 vs. 47.4%, *p* = 0.014). Compared to patients with CSPA infections, those who suffered from CRPA infections received an invasive operation (68.0 vs. 37.1%, *p* < 0.001), a tracheal intubation (60.8 vs. 34.0%, *p* < 0.001) and a blood transfusion (30.9 vs. 6.0%, *p* < 0.001) 1 month before the onset of infection. In addition, patients with CRPA infections had longer hospital stays (*p* < 0.001) and higher hospitalization expenses (*p* < 0.001) than patients with CSPA infections.

The factors mentioned above were further analyzed using binary logistic regression. The results revealed that three factors, including the length of hospitalization >28 days (OR = 3.241, 95% CI 1.622–6.473, *p* = 0.001), invasive operations (OR = 2.393, 95% CI 1.196–4.788, *p* = 0.014) and a blood transfusion (OR = 7.003, 95% CI 2.416–20.297, *p* < 0.001) within 1 month before infection, had a significant impact on the occurrence of CRPA infection. Of note, birth weight ≥2,500 g (OR = 0.278, 95% CI 0.122–0.635, *p* = 0.001) and breast nursing (OR = 0.362, 95% CI 0.168–0.777, *p* = 0.009) were significant protective factors against the development of CRPA infections in the ICUs (Table 3).

**Table 3:** Risk factors for carbapenem-resistant *Pseudomonas aeruginosa* infection with multivariate logistic regression analysis.

	***p*-Value**	**OR**	**95% CI**
Birth weight >2,500 g	0.001	0.278	0.122–0.635
Breast nursing	0.009	0.362	0.168–0.777
Length of hospitalization >28 days	0.001	3.241	1.622–6.473
Blood transfusion (within 30 days)	< 0.001	7.003	2.416–20.297
Invasive operation (within 30 days)	0.014	2.393	1.196–4.788

CRPA, carbapenem-resistant Pseudomonas aeruginosa; CSPA, carbapenem-susceptible Pseudomonas aeruginosa; OR, odds ratio; CI, confidence interval.

Bold values indicate significant p-values < 0.05.

### Risk factors for mortality of children suffering from *P. aeruginosa* infection in the ICUs

Of the 194 patients studied, 183 survived and 12 died; the overall mortality rate was 6.2% (12/194). Univariate and multivariate analyses of risk factors associated with mortality are shown in Tables 4, 5. According to the univariate analysis, none of the pediatric patients younger than 6 months died in this study. Compared to survived patients, the deceased with *P. aeruginosa* infection had a higher proportion of fever (58.3 vs. 28.0%, *p* = 0.045), septic shock (41.7 vs. 8.8%, *p* = 0.004), blood transfusion within 30 days (50.0 vs. 16.5%, *p* = 0.011), hemoglobin < 90 g/L (66.7 vs. 25.3%, *p* = 0.004), platelet < 100 × 10^9^/L (75.0 vs. 20.3%, *p* < 0.001), serum urea < 3.2 mmol/L (58.3 vs. 28.0%, *p* = 0.045) and tend to aged >3 years (75.0 vs. 17.6%, *p* < 0.001) and bacteraemia (33.3 vs. 5.5%, *p* = 0.006). Further, those who received specific antibiotic treatments before *P. aeruginosa* isolation, including aminoglycosides (41.7 vs. 9.9%, *p* = 0.007), fluoroquinolones (33.3 vs. 6.6%, *p* = 0.010), antifungal agents (50.0% vs15.9%, *p* = 0.009) are more likely had a fatal outcome. Additionally, patients who died of *P. aeruginosa* infection had higher hospitalization expenses (*p* < 0.001) than those who lived in the ICUs (Table 4).

**Table 4:** Univariate analysis for factors associated with mortality of children with *Pseudomonas aeruginosa* infection.

**Characteristics**	**Total *N* = 194**	**Survivor *N* = 182**	**Died *N* = 12**	***p*-Value**
**Demographics**				
Male gender	118 (60.8)	113 (62.1)	5 (41.7)	0.222
**Age distribution**				
< 3 months	51 (26.3)	51 (28.0)	0 (0.0)	**0.038**
3–5.9 months	29 (14.9)	29 (16.5)	0 (0.0)	0.219
6–11.9 months	42 (21.6)	41 (22.5)	1 (8.3)	0.468
12 months to 3 years	30 (15.5)	28 (15.4)	2 (16.7)	1.000
≥3 years	41 (21.1)	32 (17.6)	**9 (75.0)**	**< 0.001**
**Prematurity**	28 (14.4)	27 (14.8)	1 (8.3)	1.000
**Birth weight (g)**				
≥2,500 g	138 (71.1)	128 (70.3)	10 (83.3)	0.514
1,500–2,499 g	25 (12.9)	25 (13.7)	0 (0.0)	0.370
1,000–1,499 g	7 (3.6)	7 (3.8)	0 (0.0)	1.000
< 1,000 g	2 (1.0)	1 (0.5)	1 (8.3)	0.120
**Vaginal delivery**	102 (52.6)	94 (51.6)	8 (66.7)	0.380
**Breast nursing**	110 (56.7)	100 (54.9)	10 (83.3)	0.072
**First child**	97 (50.0)	91 (50.0)	6 (50.0)	1.000
**Intrapartum asphyxia**	16 (8.8)	16 (8.8)	0 (0.0)	0.604
**Specimen type**				
Sputum	163 (84.0)	156 (85.7)	7 (58.3)	**0.026**
Blood	14 (7.2)	10 (5.5)	4 (33.3)	**0.006**
Urine	6 (3.1)	6 (3.3)	0 (0.0)	1.000
Others	10 (5.2)	9 (4.9)	1 (8.3)	0.480
**Clinical symptoms**				
Fever	58 (29.9)	51 (28.0)	7 (58.3)	**0.045**
Icterus	17 (8.8)	16 (8.8)	1 (8.3)	1.000
Vomiting	10 (5.2)	8 (4.4)	2 (16.7)	0.120
Diarrhea	12 (6.2)	10 (5.5)	2 (16.7)	0.164
Apnea	14 (7.2)	14 (7.7)	0 (0.0)	1.000
**Complications/underlying disease**				
Severe pneumonia	38 (19.6)	38 (20.9)	0 (0.0)	0.128
Respiratory failure	14 (7.2)	14 (7.7)	0 (0.0)	1.000
Septic shock	21 (10.8)	16 (8.8)	5 (41.7)	**0.004**
Congenital heart disease	24 (12.4)	24 (13.2)	0 (0.0)	0.368
Hypoproteinemia	10 (5.2)	9 (4.9)	1 (8.3)	0.480
Anemia	26 (13.4)	25 (13.7)	1 (8.3)	1.000
Leukemia	15 (7.7)	10 (5.5)	5 (41.7)	**0.001**
**Length of hospitalization (LOS, days)**				
< 7	7 (3.6)	7 (3.8)	0 (0.0)	1.000
7–13	27 (13.9)	26 (14.3)	1 (8.3)	1.000
14–28	69 (35.6)	65 (35.7)	4 (33.3)	1.000
>28	91 (46.9)	84 (46.2)	7 (58.3)	0.553
**Antimicrobial resistance**				
CRPA	97 (50.0)	89 (48.9)	8 (66.7)	0.372
MDR-PA	83 (42.8)	76 (41.8)	7 (58.3)	0.367
XDR-PA	12 (6.2)	11 (6.0)	1 (8.3)	0.546
**Laboratory index**				
Hemoglobin < 110 g/L	86 (44.3)	86 (47.3)	0 (0.0)	**0.001**
Hemoglobin < 90 g/L	54 (27.8)	46 (25.3)	8 (66.7)	**0.004**
C-reactive protein >8 mg/L	112 (57.7)	102 (56.0)	10 (83.3)	0.076
Albumin < 35 g/L	82 (42.3)	74 (40.7)	8 (66.7)	0.128
Neutrophile >70%	70 (36.1)	69 (37.9)	1 (8.3)	0.059
Neutrophile < 50%	57 (29.4)	54 (29.7)	3 (25.0)	1.000
Lymphocyte >40%	54 (27.8)	49 (26.9)	5 (41.7)	0.320
Lymphocyte < 20%	74 (38.1)	73 (40.1)	1 (8.3)	0.032
Platelet < 100 × 10^9^/L	46 (23.7)	37 (20.3)	9 (75.0)	**< 0.001**
WBC >15.0 × 10^9^/L	46 (23.7)	44 (24.2)	2 (16.7)	0.735
ALT >69 U/L	36 (18.6)	32 (17.6)	4 (33.3)	0.241
ALT < 13 U/L	24 (12.4)	22 (12.1)	2 (16.7)	0.647
Urea < 3.2 mmol/L	58 (29.9)	51 (28.0)	7 (58.3)	**0.045**
Urea >7.1 mmol/L	39 (20.1)	36 (19.8)	3 (25.0)	0.710
Creatinine < 9 μmol/L	5 (2.6)	5 (2.7)	0 (0.0)	1.000
Creatinine >88 μmol/L	7 (3.6)	7 (3.8)	0 (0.0)	1.000
AST < 15 U/L	2 (1.0)	2 (2.1)	0 (0.0)	0.497
AST >46 U/L	106 (54.6)	97 (53.3)	9 (75.0)	0.231
**Pathogenies co-infections**				
*Acinetobacter baumannii*	19 (9.8)	18 (9.9)	1 (8.3)	1.000
*Klebsiella pneumoniae*	22 (11.3)	20 (11.0)	2 (16.7)	0.630
*Staphylococcus aureus*	8 (4.1)	7 (3.8)	1 (8.3)	0.406
*Escherichia coli*	5 (2.6)	4 (2.2)	1 (8.3)	0.276
Virus	10 (5.2)	9 (4.9)	1 (8.3)	0.480
**Treatments**				
**Previous antibiotic therapy (within 30 days)**				
3rd cephalosporins	121 (62.4)	113 (62.1)	8 (66.7)	1.000
Carbapenems	84 (43.3)	77 (42.3)	7 (58.3)	0.37
Glycopeptides	65 (33.5)	61 (33.5)	4 (33.3)	1.000
Aminoglycosides	23 (11.9)	18 (9.9)	5 (41.7)	**0.007**
Penicillins	21 (10.8)	19 (10.4)	2 (16.7)	0.623
Macrolides	23 (11.9)	22 (12.1)	1 (8.3)	1.000
Tetracyclines	9 (4.6)	8 (4.4)	1 (8.3)	0.444
Fluoroquinolones	16 (8.2)	12 (6.6)	4 (33.3)	**0.010**
Antifungal agents	35 (18.0)	29 (15.9)	6 (50.0)	**0.009**
**Blood transfusion** **(within 30 days)**	36 (18.6)	30 (16.5)	6 (50.0)	**0.011**
**Corticosteroid therapy** **(within 30 days)**	6 (3.1)	5 (2.7)	1 (8.3)	0.322
I**nvasive operation** **(within 30 days)**	102 (52.6)	94 (51.6)	8 (66.7)	0.38
Tracheal intubation	92 (47.4)	84 (46.2)	8 (66.7)	0.234
Gastric intubation	10 (5.2)	8 (4.4)	2 (16.7)	0.12
Retention catheterization	7 (3.6)	6 (3.3)	1 (8.3)	0.365
Deep venipuncture catheterization	16 (8.2)	4 (4.1)	12 (12.4)	0.065
**Parenteral nutrition**	20 (10.3)	18 (9.9)	2 (16.7)	0.357
**Previous surgery** **(within 3 months)**	91 (46.9)	87 (47.8)	4 (33.3)	0.384
**Hospitalization expenses**	122,725 (70,536–196,748)	111,366 (69,397–185,864)	327,917 (105,516–508,354)	**0.020**

CRPA, carbapenem-resistant Pseudomonas aeruginosa; CSPA, carbapenem-susceptible Pseudomonas aeruginosa; ALT, alanine transaminase; WBC, white blood cell.

Bold values indicate significant p-values < 0.05.

**Table 5:** Risk factors for mortality of *Pseudomonas aeruginosa* infection with multivariate logistic regression analysis.

	***p*-Value**	**OR**	**95% CI**
Platelet < 100 × 10^9^/L	0.044	5.729	1.048–31.308
Urea < 3.2 mmol/L	0.026	5.173	1.215–22.023

CRPA, carbapenem-resistant Pseudomonas aeruginosa; CSPA, carbapenem-susceptible Pseudomonas aeruginosa; OR, odds ratio; CI, confidence interval.

Bold values indicate significant p-values < 0.05.

On a subsequent multivariable analysis, platelet < 100 × 10^9^/L (OR = 5.729, 95% CI 1.048–31.308, *p* = 0.044) and serum urea < 3.2 mmol/L (OR = 5.173, 95% CI 1.215–22.023, *p* = 0.026) in the laboratory examination were independent risk factors for mortality due to *P. aeruginosa* infection in the ICUs. However, patients infected by CRPA did not have higher mortality rate compared with those patients with CSPA infections (Table 5).

## Discussion

CRPA infections have became a significant concern in clinical settings worldwide. Clarifying resistance trends of CRPA and related risk factors can guide appropriate antibiotic use and infection control measures. We conducted a 6-year retrospective study to evaluate the epidemiology, risk factors, and clinical outcomes of CRPA infection in ICU patients in one of the largest pediatric hospitals in China. In this study, the rates of CRPA and MDRPA among *P. aeruginosa* isolates in the ICUs were 18.4 and 25.6%, respectively, which were higher than in most countries, but only lower than in India (29.3 and 28.2%) and China (27.1 and 29.9%) from surveillance data for the Asia-Pacific countries (23). Furthermore, more than 90% of CRPA strains in our hospital were isolated from ICU wards. Therefore, continued infection control and appropriate antibiotic use are important to maintain decreases in CRPA infections.

Previous reports have shown the length of hospitalization and receiving invasive procedures were often regarded as risk factors associated with CRPA infection (24–26). Similar to some other studies, our study also found a positive correlation between the incidence of CRPA infection and the length of hospital stay prior to CRPA confirmation. An ICU is a special ward where critically ill patients are admitted, who are vulnerable to repeat or opportunistic infections, who stay longer in the ICU and are longer exposed to several antibiotics (26). A retrospective cross-sectional research in Taiwan discovered patients had a 1% increased risk of CRPA infection with an extra day in the hospital (26). Furthermore, ICU patients tend to undergo surgery and invasive medical procedures more frequently than general patients, which compromise their immune systems and defense barriers of the skin and mucosa; this provides a pathway for CRPA to enter the injured mucous membrane and cause serious infection (24). Apart from the length of hospitalization and invasive procedures, blood transfusion was also a significant risk factor associated with the occurrence of CRPA infection. Blood transfusion remains a common treatment in ICU patients, which carries significant risks, including risks for infectious agent transmission and immunosuppression (27). In mainland China, blood products are thoroughly screened to avoid transfusion-transmissible infections. The increased risk of CRPA infection may be the result of transfusion-induced immunosuppression, which has previously been associated with the increased activity of suppressor T cells and decreased activity of natural killer cells (28). Therefore, unnecessary transfusions should be avoided to reduce the occurrence of CRPA infection.

Breastfeeding offers important health benefits and protection for babies, such as better gastrointestinal function and reduced risk of respiratory infections (29). Early breastfeeding has been reported to significantly reduce the risk of nosocomial sepsis in the NICU (30). The efficacy of breast milk appears to be dose dependent in NICU and the incidence of any infection including meningitis was significantly decreased (31). It supports this assertion that early breastfeeding should be considered to be an important prevention strategy for nosocomial infections in NICUs. In addition, lower birth weight (LBW) was one of the most common risk factors related to CR-GNB infection in neonates/infants. More interestingly, the incident rate of late-onset sepsis (LOS) was inversely associated with birth weight (32). According to a Peruvian study, the incidence of LOS was high in very LBW infants, reaching 36.2% and even higher in extremely LBW infants (40.7%) (33). Further, LBW (< 2,000 g) was an important risk factor affecting the mortality of sick newborns with multidrug resistant gram-negative infections (34). On the contrary, birth weight ≥2,500 g was a significant protective factor against infection and death caused by drug-resistant strains.

The prevalence of CRPA strains presents severe challenges for treating *P. aeruginosa* infections, with antibiotic resistance associated with a worse prognosis. In our study, the crude in-hospital mortality of *P. aeruginosa* infection was 6.2%, which is considerably less than many other studies. The mortality of patients ranged from 18 to 62% due to *P. aeruginosa* bacteraemia (35). A meta-analysis of 6,284 individual patients from 24 trials found the overall attributable mortality of ventilator-associated pneumonia was 13% (36). It highlighted the notable difference in the mortality of *P. aeruginosa* isolates among different sources and locations. Our study also found *P. aeruginosa*-infected survivors mainly came from sputum (*p* = 0.026), while those infected non-survivors were mostly from blood (*p* = 0.006).

Platelets are the smallest blood cells that play an important role in linking the hemostatic and inflammatory processes in the body. Recent discoveries have revealed platelet expression of Toll-like and purinergic receptors, which are able to detect and respond to bacterial infections, degrade a range of antimicrobial peptides, and coordinate immune systems against bacteria (37). This is in agreement with the finding that abnormalities in platelet quality or quantity predisposes individuals to greater risk or severity of microbial infection. Lippi et al. reported thrombocytopenia was related to a three-fold enhanced risk of severe COVID-19 (38), similar results occurred in our study. To the best of our knowledge, this is the first study to show that low platelets is a significant predictor of mortality due to CRPA infections.

Serum urea is commonly used as a clinical indicator of renal function in healthcare settings. Urea is the end product of protein catabolism in mammals, which is synthesized by the ornithine cycle in the liver and excreted mainly by the kidneys. Serum urea concentrations are highly variable and depend, in addition to renal function, on the liver and cardiac functions, dietary protein intake, metabolic rate, medication status, and hydration status (39). These factors are common in critically ill patients in the ICUs, which supports that the patient's physical condition is closely related to their survival. This supports the finding that serum urea is a significant risk factor for mortality in patients with CRPA infections.

Our study has some limitations. Firstly, it was single-center retrospective research, under-reporting may occur in some cases. Secondly, the limited sample size of this study might have influenced the power of the analysis to distinguish significant factors associated with CRPA infection or mortality. Lastly, data on genotypes and/or mechanisms of resistance of the CRPA isolates was not available, which would have provided important information for tracing the source of infection. It should be included in further surveillance studies.

In summary, our study has revealed that longer hospital stays (>28 days), invasive operations and a blood transfusion (within 1 month before infection) were independent risk factors for the development of CRPA infections among pediatric patients in the ICUs. Of note, adequate birth weight (≥2,500 g) and breast nursing were significant protective factors against CRPA infections. Contrary to most earlier studies, mortality was not linked with carbapenem resistance in our study. Reduced platelet counts and serum urea were significant predictors of mortality in critically ill children with *P. aeruginosa* infections.

## Data availability statement

The original contributions presented in the study are included in the article/supplementary material, further inquiries can be directed to the corresponding author.

## Ethics statement

The studies involving human participants were reviewed and approved by IRB of Shanghai Children's Medical Center Affiliated to Shanghai Jiaotong University, School of Medicine. Written informed consent from the participants' legal guardian/next of kin was not required to participate in this study in accordance with the national legislation and the institutional requirements. Written informed consent was not obtained from the individual(s) for the publication of any potentially identifiable images or data included in this article.

## Author contributions

XWa contributed to study conception, study design, manuscript drafting, and critical manuscript revision. All authors contributed to data collection, data analysis, and critical revising the paper, have agreed on the journal to which the article will be submitted, gave final approval of the version to be published and agree to be accountable for all aspects of the work.
